# Adaptive expansion of the maize *maternally expressed gene* (*Meg*) family involves changes in expression patterns and protein secondary structures of its members

**DOI:** 10.1186/s12870-014-0204-8

**Published:** 2014-08-01

**Authors:** Yuqing Xiong, Wenbin Mei, Eun-Deok Kim, Krishanu Mukherjee, Hatem Hassanein, William Brad Barbazuk, Sibum Sung, Bryan Kolaczkowski, Byung-Ho Kang

**Affiliations:** 1 Department of Microbiology and Cell Science, University of Florida, Gainesville 32611, FL, USA; 2 Department of Biology, University of Florida, Gainesville 32611, FL, USA; 3 Department of Molecular Biosciences and Institute for Cellular and Molecular Biology, University of Texas at Austin, Austin 78712, TX, USA

**Keywords:** ■■■

## Abstract

**Background:**

The *Maternally expressed gene* (*Meg*) family is a locally-duplicated gene family of maize which encodes cysteine-rich proteins (CRPs). The founding member of the family, *Meg1*, is required for normal development of the basal endosperm transfer cell layer (BETL) and is involved in the allocation of maternal nutrients to growing seeds. Despite the important roles of *Meg1* in maize seed development, the evolutionary history of the *Meg* cluster and the activities of the duplicate genes are not understood.

**Results:**

In maize, the *Meg* gene cluster resides in a 2.3 Mb-long genomic region that exhibits many features of non-centromeric heterochromatin. Using phylogenetic reconstruction and syntenic alignments, we identified the pedigree of the *Meg* family, in which 11 of its 13 members arose in maize after allotetraploidization ~4.8 mya. Phylogenetic and population-genetic analyses identified possible signatures suggesting recent positive selection in *Meg* homologs. Structural analyses of the Meg proteins indicated potentially adaptive changes in secondary structure from α-helix to β-strand during the expansion. Transcriptomic analysis of the maize endosperm indicated that 6 *Meg* genes are selectively activated in the BETL, and younger *Meg* genes are more active than older ones. In endosperms from B73 by Mo17 reciprocal crosses, most *Meg* genes did not display parent-specific expression patterns.

**Conclusions:**

Recently-duplicated *Meg* genes have different protein secondary structures, and their expressions in the BETL dominate over those of older members. Together with the signs of positive selections in the young *Meg* genes, these results suggest that the expansion of the *Meg* family involves potentially adaptive transitions in which new members with novel functions prevailed over older members.

## Background

Transfer cells in plants mediate solute transport between the apoplast and the symplast. One structural feature of plant transfer cells is the extensive secondary cell wall growth, which increases the plasma membrane surface area and is thought to facilitate rapid solute transport across the plasma membrane [1]. In agreement with their solute exchange activity, transfer cells are typically observed in sink or source tissues in the vicinity of vascular tissues. At the base of the maize endosperm, a layer of transfer cells faces the maternal placento-chalazal zone [2]. Seed development in maize is dependent on nutrient transfer through this cell layer, termed the basal endosperm transfer cell layer (BETL).

Cysteine rich proteins (CRPs) constitute a large superfamily of small, secreted proteins abundant in eukaryotes [3],[4]. CRPs are involved in both cell-signaling [5],[6] and antimicrobial processes [7]. In plants, cell-cell communications mediated by secreted CRPs contribute to stomata differentiation [8], to guiding pollen tube growth [9] in self-incompatibility [10], and patterning embryo development [11]. BETL in the maize endosperm also secretes multiple types of CRPs, including *basal endosperm transfer layer1 (BETL-1)*, *2 (BETL-2)* and *4 (BETL-4)*[12], *BAP*[13], and *maternally expressed gene 1* (*Meg1*) [14]. It was shown that a MYB-like transcription factor that plays a key role in BETL development, ZmMRP-1, is involved in expression of *BETL-1*, *BETL-2*, and *Meg1*[14]–[17]. Given that the BETL is at the maternal-filial interface, these CRPs may protect developing seeds from maternally-transmitted pathogens [18]. It is also possible that some BETL CRPs serve as extracellular signal molecules that coordinate the supply of maternal nutrients during seed development [3].

The *Meg1* gene is required for normal development of the BETL, and elevated expression of *Meg1* increases BETL sizes and seed biomass. Interestingly, ectopic expression of *Meg1* drives the expression of BETL-specific genes such as ZmMRP-1 and INCW2 in non-BETL endosperm cells. Because *Meg1* is a maternally expressed imprinted gene, and the effects of *Meg1* are dosage dependent, the promotion of nutrient uptake by *Meg1* provides evidence that nutrient uptake during seed development is under maternal control [19],[20]. The enhanced nutrient allocation resulting from *Meg1* over-expression suggests that the Meg1 protein contributes to establishing the sink strength of developing seeds by controlling BETL. A group of CRPs, termed Embryo Surrounding Factor 1 (ESF1), play roles similar to Meg1 in Arabidopsis. The suspensor at the base of the embryo is involved in nutrient transport in Arabidopsis and ESF1s produced from the central cells and endosperm cells promote suspensor development [11].

Homologs of *Meg1* are also transcribed in the developing endosperm [14]. We have shown that these *Meg1* homologs are among the most highly-expressed genes in the BETL [21]. The existence of active *Meg1* homologs raises questions about how this family arose and whether various *Meg1* homologs play similar or different functional roles. In this study, we identify the global complement of functional and non-functional *Meg* family genes in maize and in the closely-related sorghum outgroup; we use a combination of phylogenetic and population-genetic techniques to characterize selection pressures across these genes and link selection to changes in gene expression and protein structure. We find that the *Meg* gene family expanded rapidly in maize, with some evidence suggesting that positive selection may have driven changes in protein structure. Our analysis indicates that more recent duplicates exhibit higher expression levels, more extensive structural changes, and stronger evidence for adaptation than do older duplicates, suggesting that newer, functionally different *Meg* homologs may have prevailed over older homologs during recent adaptation.

## Results and discussion

### Identification of *Meg* genes in maize

The *Meg1* gene in maize is a member of the large Meg/Ae1 supergroup of CRPs consisting of 17 subgroups sharing a simple CXCC motif but little detectable sequence similarity [4]. We focused our attention on the subgroup CRP5420, which includes *Meg1* and other members containing the cysteine motif: CX(6)CX(4)CYCCX(14)CX(3)C and exhibiting conserved amino acid sequence. Based on sequence conservation, we identified 13 loci in the B73 maize genome homologous to *Meg1*, including *Meg2, Meg3, Meg4, and Meg6* that have been identified previously together with *Meg1*[14]. The B76 genome does not contain any open reading frame that matches *Meg5*. We named 8 new members *Meg7*—*Meg14* according to their chromosome position. The seven loci upstream of *Meg1* were named *Meg7*—*Meg13* from proximal to distal to the *Meg1* gene, and the locus downstream of *Meg1* was named *Meg14* (Additional file 1: Table S1).

**Figure 1:**
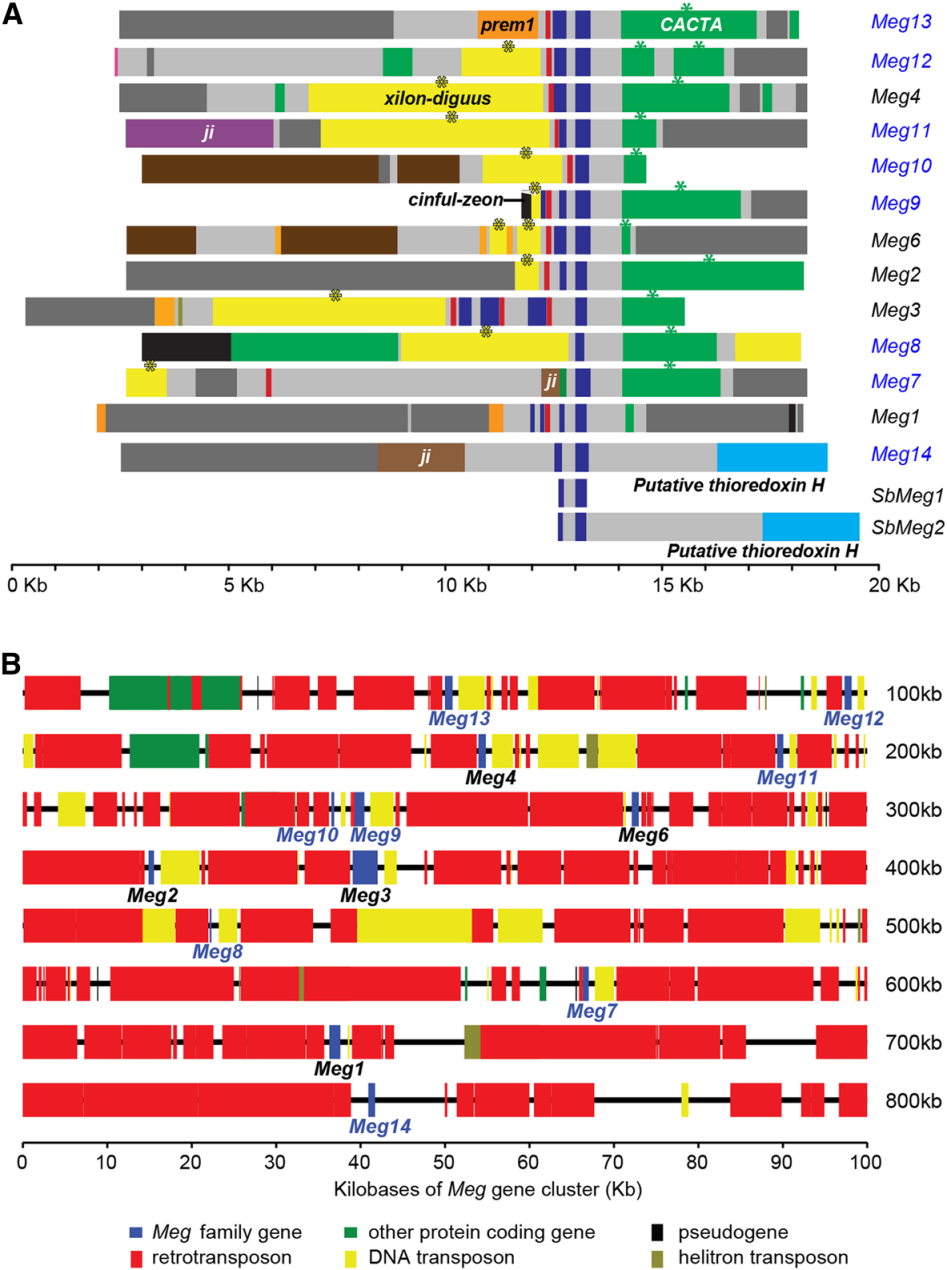
**Gene structures and genomic arrangement of the 13*Meg*genes in maize. (A)***Meg* genes and their flanking regions are aligned to illustrate their gene structures. Promoters and exons of *Meg* genes are depicted as red and blue rectangles, respectively. Note that *Meg14* is missing the canonical *Meg* promoter. Each superfamily of transposons is shown as a rectangle with the following color codes: *xillon-digus -* yellow, *prem1* - orange, *ji* - brown. The transposon insertions within 10 kb upstream and 5 kb downstream of each gene model are shown. All of the *Meg* genes except *Meg1*, *Meg13* and *Meg14* have xillon-digus on their 5’ side and CACTA sequences on their 3’ side (asterisks). Two putative H-type thioredoxins downstream of *Meg14* and *SbMeg2* are colored light blue. All other regions are colored gray. All components of the region were drawn to scale according to their physical sizes. **(B)** The 800 kb region in chromosome 7S that contains the 13 *Meg* genes is detailed. Color codes for the 6 main elements in the region are provided under the diagram.

The *Meg1* gene consists of two coding exons separated by a single intron and an upstream promoter required for specific expression in basal endosperm transfer cells (BETCs) [14]. We found that the complete *Meg1* gene architecture is shared by 8 *Meg* homologs (Figure 1A). Exceptions were *Meg7*, *Meg8*, *Meg3*, *Meg10* and *Meg14. Meg14* has the two canonical exons but its promoter is distinct from that of *Meg1*. The first coding exon is missing from *Meg10* and *Meg8. Meg8* does not appear to have promoter elements, suggesting that it may not be transcribed. The flanking sequences of *Meg8* and *Meg10* suggest that disruption of the two genes has been caused by non-homologous end joining. *Meg7* has the two coding exons, but its promoter is dislocated ~6.2 kb upstream from the first exon by a transposon insertion. The structure of *Meg3* is abnormal in that it has multiple regulatory elements and extra exons that are disarranged.

### Clustering of maize *Meg* genes

All 13 *Meg* loci reside on maize chromosome 7S, between the molecular markers *p-asg8* and *p-asg34*. When compared with chromosome regions where gene density is high or where local gene duplicates are concentrated, this *Meg* region exhibits several distinct features. First, rather than tightly clustering in a genic island like other maize gene clusters [22], the thirteen loci of the *Meg* family are spread over a genomic region of ~800 kb (Figure 1B). Also gene density is lower in the *Meg* region than in other genic regions of the maize genome; the average distance between neighboring *Meg* genes is 62 kb, larger than the average interval between similar locally-duplicated genes such as *p1*, *rp1*, *zein*, *kn1*, *pl1*, *a1-b*, or *rp3* (Additional file 2: Table S2). The density of genes in the *Meg* cluster is even lower than the average gene density of the entire maize genome (one gene/52 kb, based on the filtered gene content of the 2066 Mb RefGen_v2 whole genome assembly in http://maizesequence.org/). The overly-dispersed nature of the *Meg* gene cluster is striking, considering the general tendency of maize genes to concentrate in tightly-integrated gene islands [22].

Approximately 85% of the maize genome consists of transposable elements, with *gypsy* transposons tending to predominate in gene-poor heterochromatic regions [23] and *Mutator* transposons tending to predominate in genic regions and in open chromatin [24]. In contrast this general pattern across the maize genome, *gypsy* transposons comprise 75% lengthwise of all transposable elements in the 800-kb *Meg* region, and *Mutator* transposons are completely absent from this region (Figure 1B).

Chromosomal recombination tends to occur often in euchromatin but is suppressed in heterochromatin [25]. Consistent with the presence of the *gypsy* heterochromatic-marker transposons and highly-dispersed genes, the 2.3-Mb genomic region containing the *Meg* cluster (from 10.85 to 13.86 Mb of chromosome 7S) shows a low recombination rate of < 1 centimorgan (cM) (Liu et al. [24]; http://www.maizegdb.org). The 3.3-Mb region upstream (from 7.38 to 10.68 Mb) and the 3.7-Mb region downstream (13.92—17.05 Mb) flanking the low-recombining *Meg* region represent ~15.8 and ~8.5 cM of genetic distance, respectively, suggesting that the region surrounding the *Meg* gene cluster represents a localized region of reduced recombination. Taken together, these data suggest that the *Meg* gene region displays characteristics of maize non-pericentromeric heterochromatin.

We found that all members of the *Meg* cluster, except *Meg1* and *Meg14,* are surrounded by homologous 5’ and 3’ flanking sequences (Figure 1A). The lengths of the homologous flanking sequences vary from a few hundred base pairs to more than 5 kb. The 5' flanking sequences of nine genes (*Meg2, 3, 4, 6, 8, 9, 10, 11, and 12*) contain *xilon-digus* retrotransposons, which vary in length. In contrast, *Meg13* and *Meg1* have *prem1* retrotransposon insertions at the beginning of their 5' flanking sequences. The 3' flanking sequences of all *Meg* genes, except *Meg14,* are homologous. *Meg14* is peculiar in that the flanking sequences on both sides are not homologous to any of the other 12 *Meg* genes, suggesting that it may have a unique origin. The general homology of the sequences surrounding the *Meg* genes suggests that expansion of the *Meg* family can be primarily attributed to unequal crossover and insertion of transposable elements that left characteristic signatures up- and down-stream of duplicate genes.

### Evolutionary history of *Meg* genes

The *Meg* gene cluster resides exclusively on chromosome 7S in maize. We searched the public databases to identify homologs of *Meg* genes in other grass species. Two open reading frames in sorghum (*Sorghum bicolor*) displayed strong sequence similarity with *Meg1* and other members of the maize *Meg* gene cluster, and one gene in foxtail millet (*Setaria italica*) was identified as a potential homolog. We found no homologs in rice or other closely-related species, suggesting that *Meg* genes originated before the sorghum/maize split but after the Panicoideae group diverged from other grass species [PMID: 22580950]. Although Meg1-related peptides of Arabidopsis, ESF1s, have been identified and functionally characterized [11], there is no detectable sequence similarity between *ESF1s* and the genes identified in maize and other grass species, asides from their conserved patterns of cysteine residues. Short secreted peptides such as *Meg* typically evolve very rapidly, making the determination of precise phylogenetic relationships across large timescales difficult. We therefore restricted our analyses to those *Meg* homologs displaying reliable sequence similarity, although the actual evolutionary origin of this gene family is likely to have been much earlier.

Using sequence similarity to *Meg* genes and to other genes flanking the maize *Meg* cluster, we identified regions in the maize, sorghum, and rice genomes that are homologous or homeologous to the 800-kb *Meg*-containing region. The maize *Meg* genes and their sorghum homologs reside exclusively in a syntenic block conserved throughout grass genomes (Additional file 3: Figure S1). Gene colinearity is well-retained in the syntenic blocks of maize, sorghum and rice, although the 4-Mb region of maize chromosome 7S containing the *Meg* genes is five times larger than the corresponding region in rice, which lacks *Meg* homologs. The complete lack of *Meg* genes in the homeologous region of maize chromosome 2 suggests that the duplication events in the *Meg* family happened only in chromosome 7, primarily after allotetraploidization ~4.8 million years ago (mya) [26],[27], while the *Meg* copies in chromosome 2 were lost.

In order to confirm that the expansion of the *Meg* gene family is not an anomaly of the B73 inbred line, we estimated copy numbers in six additional maize cultivars. All *Meg* loci were amplified from each cultivar, and amplicons were sequenced to determine whether the specific polymorphisms in each *Meg* gene were present in the amplicons (Additional file 3: Figure S2). With few exceptions, all six inbred lines share the complete complement of *Meg* genes, suggesting that *Meg* gene family expansion probably occurred before the establishment of modern maize cultivars. Further supporting this hypothesis, we were able to confirm all the *Meg* homologs from teosinte (*Zea mays ssp. parviglumis*), suggesting that the *Meg* gene cluster had fully expanded before maize was domesticated from its wild ancestor, ca. 4000–10,000 years ago (Additional file 3: Figure S2).

**Figure 2:**
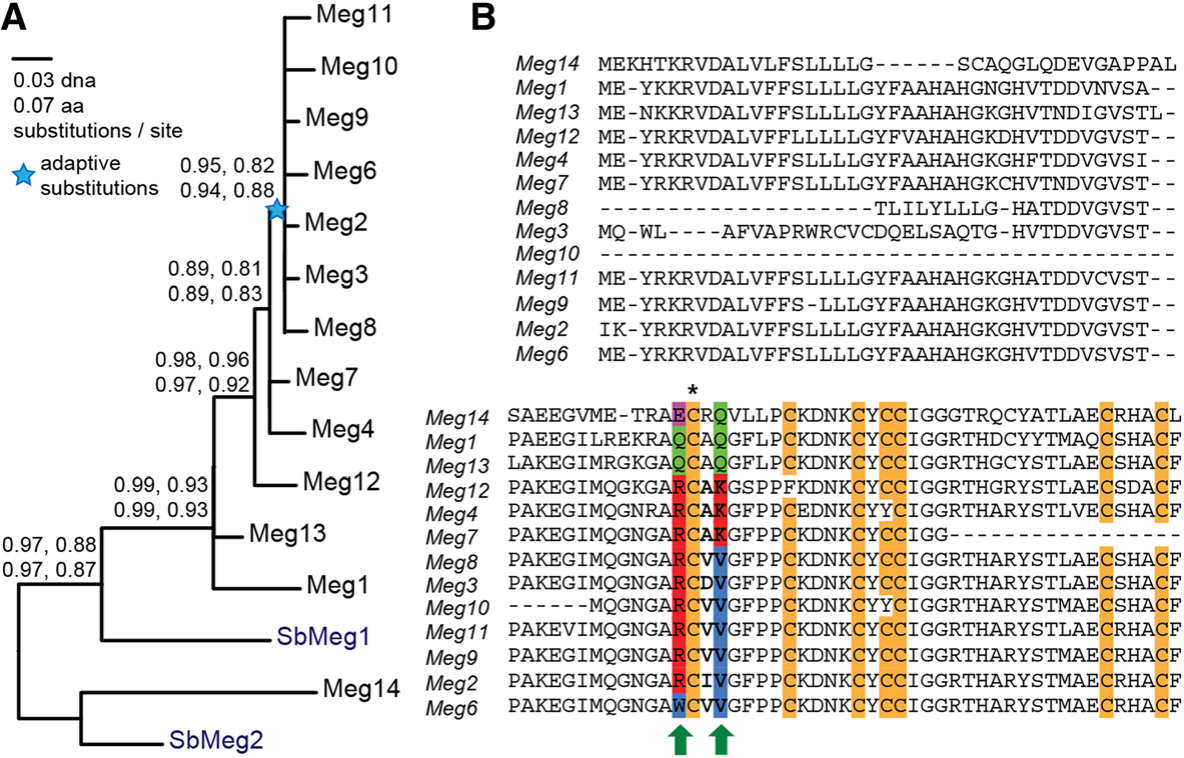
**Phylogenetic analyses of maize*Meg*genes identifies adaptative amino acid substitutions. (A)** We reconstructed maximum likelihood phylogenies from protein and corresponding DNA sequence data. SH-like aLRT support [28] at key nodes is shown for protein sequence data with and without Gblocks [29] processing to remove unreliable alignment positions (top row) and DNA alignments with and without Gblocks processing (bottom row). Nodes having <0.8 SH-like aLRT support in any analysis are collapsed, and the tree is rooted using gene-species tree reconciliation to minimize duplication/loss events. A blue star indicates significant support for adaptative substitutions in that specific branch (*p* < 0.05 after correcting for multiple tests), inferred using codon-based analysis (see Methods). **(B)** We plot amino-acid substitutions inferred as adaptive by branch-sites analysis (Zhang *et al*) [30] along the alignment of Meg protein sequences (green arrows). Biochemical properties of amino acids are marked as pink for hydrophilic polar, green for hydrophilic polar uncharged, red for hydrophilic polar basic, and blue for hydrophobic nonpolar amino acids. Conserved cysteine residues are highlighted in orange.

We reconstructed the phylogeny of *Meg* family genes using maximum likelihood, with the distantly-related foxtail millet *Meg* gene used as an outgroup. The resulting phylogeny identified a large clade consisting of the 12 B73 *Meg* genes and one of the sorghum *Meg* homologs (*SbMeg1*), separated from *Meg14* and the other sorghum homolog (*SbMeg2*) with strong statistical support (Figure 2A). Maize *Meg14* and sorghum *SbMeg2* share homologous downstream flanking sequences and a nearby putative thioredoxin H gene (Figure 1A), further supporting their grouping. Together, these data suggest that maize *Meg14*/*SbMeg2* may have diverged from the maize *Meg1-13*/*SbMeg1* clade after the maize/sorghum group split from millet but prior to the maize/sorghum divergence.

In addition to outgroup rooting using the foxtail millet *Meg* sequence, we used gene-tree/species-tree reconciliation to estimate the rooted phylogeny by minimizing gene gain/loss events [31]. The most parsimonious rooting (Figure 2A) supports the view that two *Meg* genes were present in the common ancestor of maize and sorghum. One of these ancestral genes was retained as a single copy in both species (maize *Meg14*/*SbMeg2*), while the other ancestor underwent a series of at least two rapid expansions in the maize genome. Maize *Meg1* falls at the base of the maize-specific expansion and is separated from the other *Meg* homologs with strong support. *Meg1* is also located downstream from the other maize-specific *Meg* genes (Figure 1A), suggesting that the *Meg1* gene was probably the original progenitor of the maize expansion that would have occurred through a series of “upstream” duplication events. The consistency between phylogenetic “age” and chromosome position supports this general model, with genes closer in physical location to *Meg1* tending to fall toward the base of the *Meg* phylogeny (see Figures 1B and 2A).

To date the time of *Meg* gene duplications, we reconstructed the maximum likelihood phylogenetic tree using a molecular clock calibrated with a maize-sorghum divergence time of ~11.9 mya [26]. Consistent with the absence of *Meg* genes on maize chromosome 2, molecular-clock analysis suggested that *Meg* gene expansions occurred after maize allotetraploidization (Additional file 3: Figure S3). According to this analysis, the majority of *Meg* genes (*Meg2-11*) appeared very recently through a rapid series of duplication events that cannot be resolved phylogenetically (i.e. approximately 0.90—1.58 mya). *Meg12* was inferred to have arisen ~1.77—2.77 mya, and the oldest duplicates following the maize-sorghum split, *Meg1* and *Meg13*, arose ~3.07—4.80 mya, right after maize allotetraploidization. Although we are cautious in our assignment of concrete dates to these duplication events, as molecular-clock assumptions are likely to be violated, these results suggest a model in which the *Meg* gene cluster expanded rapidly in maize after allotetraploidzation (~4.8 mya) but before domestication (~4000-10,000 years ago). These results are corroborated by examination of synteny and phylogenetic analyses (Figure 2A, Additional file 3: Figure S1), which do not rely on molecular-clock assumptions.

### Evidence for positive selection driving changes in *Meg* protein secondary structure

Functional divergence of cysteine rich proteins (CRPs) has often been linked to gene duplication followed by positive selection acting to alter protein function [32]–[34]. We used statistical analyses based on examining the ratio of nonsynonymous to synonymous substitutions in order to characterize the possible role of adaptive processes in shaping the protein functions of maize *Meg* homologs. These analyses identified a single branch on the phylogeny as exhibiting strong evidence for protein-coding adaptation, the branch uniting *Meg3-9*, which represents the most recent maize-specific expansion event (*p* < 0.05 after correcting for multiple tests; Figure 2A).

Branch-sites analysis further identified two amino-acid substitutions on the *Meg3-9* branch that appear to have been driven by positive selection (Figure 2B). These substitutions replace a conserved AK motif next to the first conserved cysteine with a VV motif, altering the size, charge and hydrophobicity of this region. An additional unusual Arg to Trp substitution in *Meg6* in front of the same cysteine residue suggests that this position may represent a “hotspot” of Meg protein functional differentiation.

**Figure 3:**
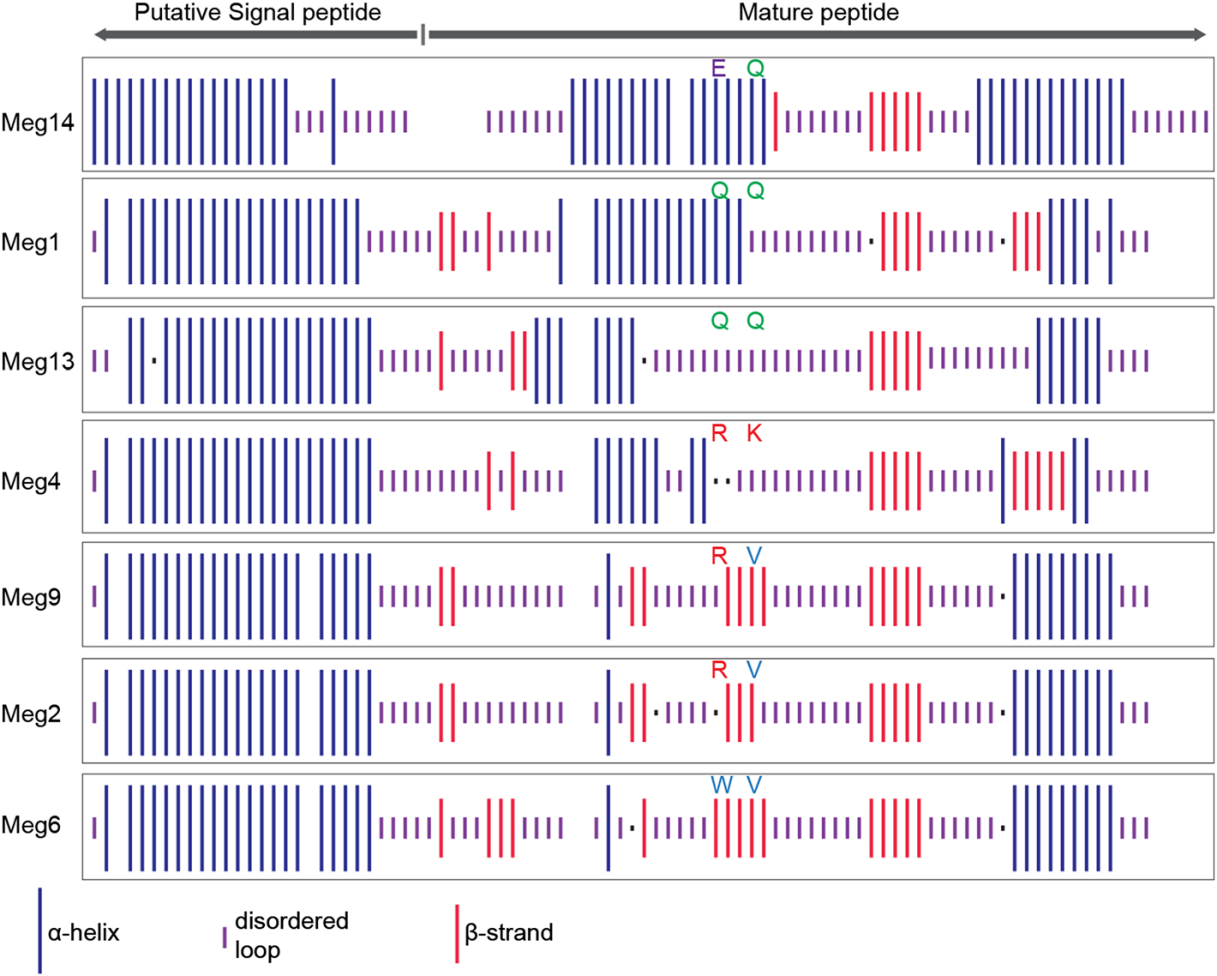
**Meg protein secondary structure has changed over the maize-specific gene family expansion.** The secondary structures of Meg proteins were predicted using different algorithms on the Network sequence analysis server (NPS@, Network Protein Sequence Analysis, http://npsa-pbil.ibcp.fr). The α-helix, β-strand and disordered loop regions are denoted by the longest, the second longest and the second shortest bars, respectively. The shortest bars represent residues with ambiguous states. The symbols of positively selected amino acids are shown above the corresponding bars. Gaps were introduced according to the amino acid sequence alignment in order to align secondary structural elements for visualization. The figure illustrates amino acid sequences of *Meg* genes whose coding sequences are intact.

Although crystal structures to support homology modeling of Meg proteins are not available, we characterized secondary structures of Meg proteins to identify possible structural consequences of amino-acid substitutions. We found that there was a general reduction in the proportion of α-helices and a corresponding increase in β-strands during the maize-specific *Meg* family expansion (Table 1, Figure 3). For example, the oldest Meg proteins, Meg1 and Meg14, were predicted to contain 52.81% and 45.45% α-helices, respectively. In contrast, the youngest proteins, Meg9, Meg2 and Meg6, were 35.63%, 36.36% and 36.36% alpha-helix, respectively (Table 1). The alpha-helix content of the evolutionary intermediates, Meg13 and Meg4, fell between those of the oldest and youngest genes (*i.e.* 38.64% and 37.50%, respectively). Proportions of β-strand displayed the opposite trend, with β-strand proportion increasing from oldest to youngest (Table 1).

**Table 1:** **Composition of secondary structures in** ***Meg*** **proteins**

**Types of secondary structure**	**α-helix**	**β-strand**	**Random coils**
Meg14	52.81%	6.74%	40.45%
Meg1	45.45%	11.36%	40.91%
Meg13	38.64%	9.09%	50.00%
Meg4	37.50%	13.64%	46.59%
Meg9	35.63%	14.94%	48.28%
Meg2	36.36%	13.64%	46.59%
Meg6	36.36%	17.05%	44.32%

We are cautious in our interpretation of secondary-structure predictions, as modern methods only achieve ~80% accuracy [http://ieeexplore.ieee.org/xpls/abs_all.jsp?arnumber=6217208]. However, it is interesting to note that localized changes in predicted protein secondary structure correlate strongly with the specific amino acids identified as being under positive selection (Figure 3). This protein region forms the first α-helix of the mature peptide in Meg1 and Meg14. The region surrounding the adaptive changes is predicted as disordered in the intermediate-aged Meg4 and Meg13, leading to an overall reduction in the length of this first α-helix. In the more recently derived Meg2, Meg6, and Meg9, the first α-helix is predicted as completely missing and is replaced by a conserved β-strand (Figure 3). Overall, these results suggest that the N-terminal region of maize Meg proteins has undergone a systematic and directional structural reorganization throughout the expansion of the *Meg* gene family. Although the absence of 3D structural data and the low accuracy of secondary structure prediction limit our ability to draw strong conclusions about how changes in Meg protein sequence may have changed protein function, the confluence of adaptive protein-coding changes and alteration of predicted secondary structures do suggest that these evolutionary changes have altered Meg protein function in some way.

### Evidence for recent selective sweeps in the maize *Meg* gene cluster

**Figure 4:**
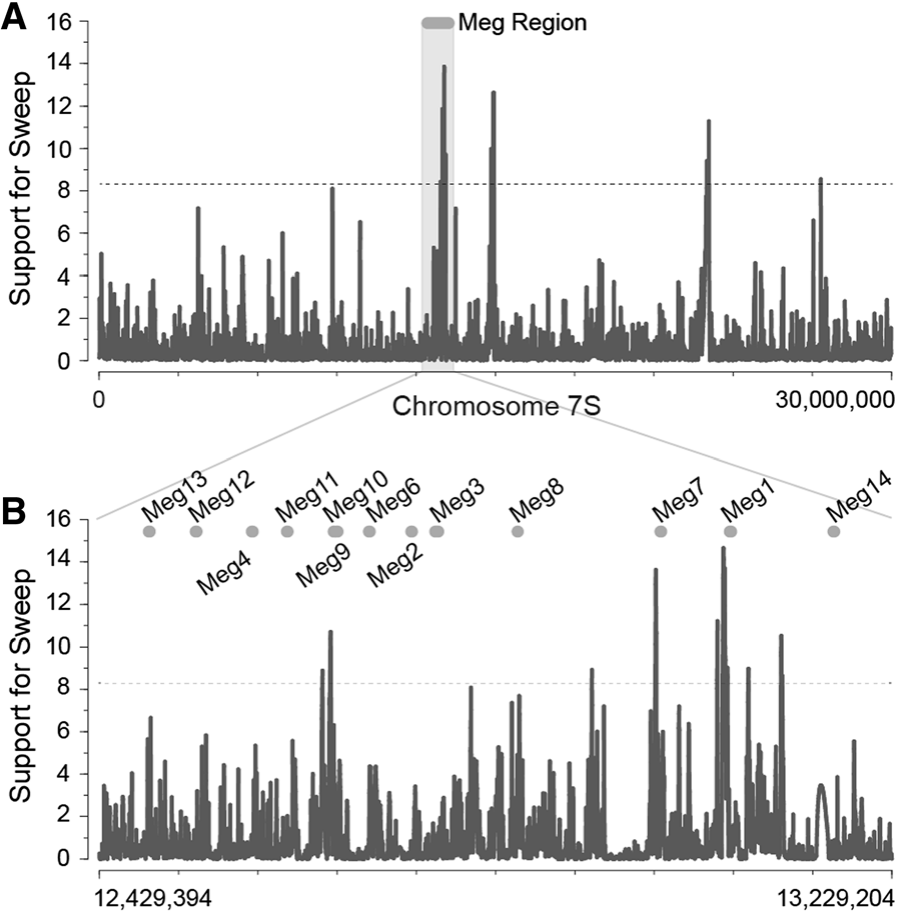
**Selective sweeps in maize*Meg*gene region identified by composite-likelihood analysis.** We used a spatially-explicit likelihood model to identify recent selective sweeps within the region of maize chromosome 7S containing the *Meg* gene array from polymorphism data (see Methods). We plot the log-likelihood support in favor of a selective sweep model along chromosome position. A dotted horizontal line indicates the empirically-derived 0.05 significance cutoff, with log-likelihood greater than the dotted line indicating significant support for a selective sweep. **(A)** We plot support for a selective sweep across the 30-Mb region of chromosome 7S containing the *Meg* gene region. **(B)** Close-up of the chromosomal region containing the *Meg* gene cluster, with each *Meg* gene’s coding sequence indicated.

To investigate the possible role of recent selective sweeps in maize *Meg* gene evolution, we analyzed maize polymorphism data [35],[36] using a composite-likelihood method to identify population-level adaptation [37]. We found that the *Meg* region had the strongest signature of an adaptive sweep across the entire distal 30 Mb of maize chromosome 7S (Figure 4A). Although we are cautious about the ability of these methods to identify the precise locations of selective sweeps across the genome [37], we note that the strongest support for population-level adaptation localized to *Meg9—10* and just upstream of *Meg1* and *Meg7* (Figure 4B). The functional consequences of these putative adaptive sweeps remain unknown, although these results do suggest that the maize *Meg* gene cluster may have experienced recent positive selection, further supporting a general model of maize adaptation through *Meg* gene family expansion and diversification.

It is impossible to draw definitive conclusions about adaptive changes in protein function from phylogenetic and population-genetic analyses, alone so we consider these conclusions speculative at this point. However, we note that the combination of statistical evidence for elevated nonsynonymous/synonymous substitution ratios, nonconservative amino-acid substitutions, localized changes in predicted secondary structure, and population-genetic evidence for possible selective sweeps all argue in favor of a model in which adaptation has played a role in the maize *Meg* gene expansion.

### Expression profiles of *Meg* genes

**Figure 5:**
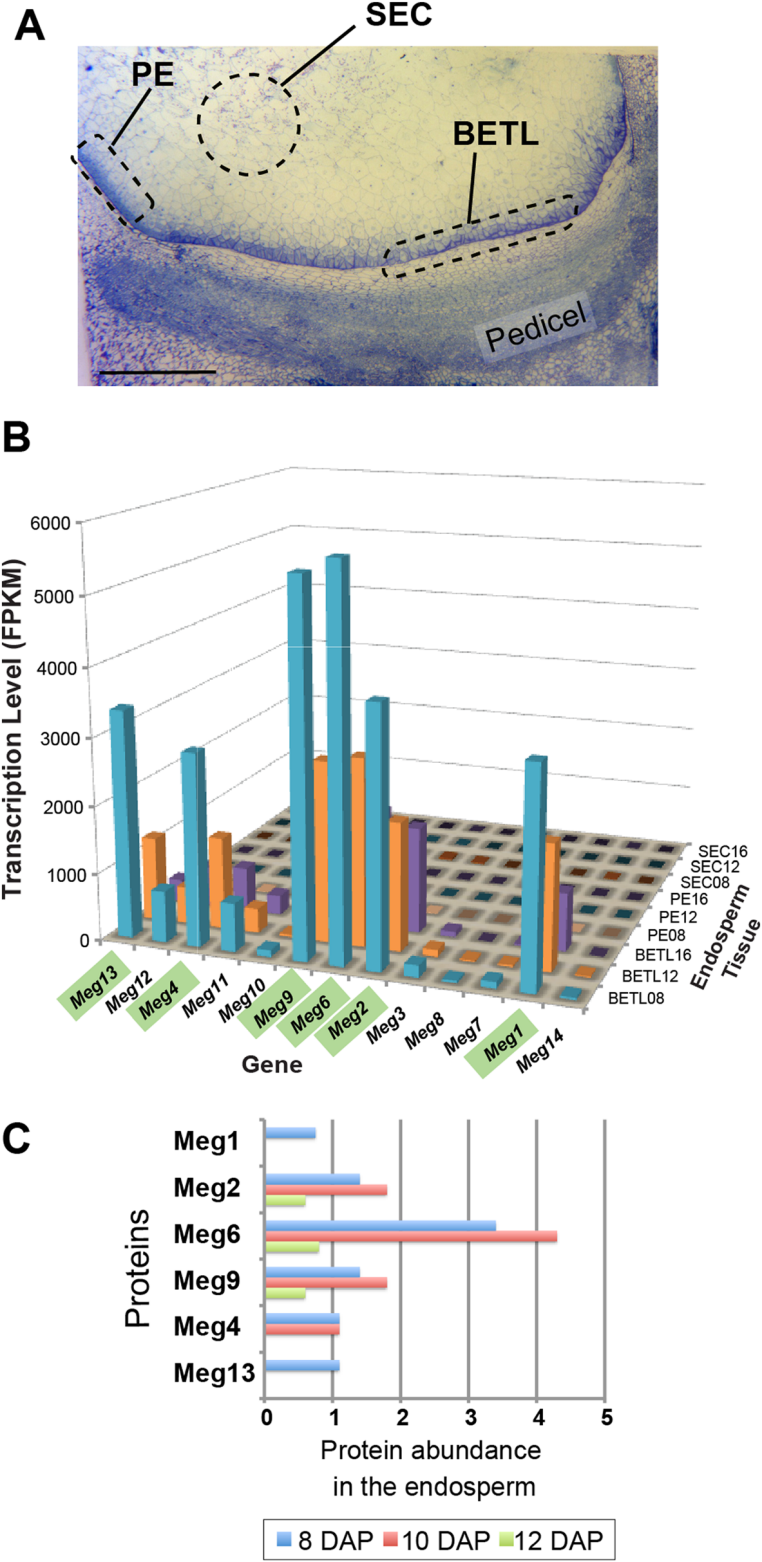
**Specific*Meg*homologs are highly expressed in maize endosperm. (A)** Bright-field micrograph of a maize endosperm at 8 days after pollination (DAP), showing the basal endosperm transfer cell (BETC), peripheral endosperm (PE) and starchy endosperm cell (SEC) layers. These three tissue types were isolated by cryo-microdissection, and gene-specific transcripts were evaluated by RNA-seq. Scale bar: 0.5 ○m. **(B)** Transcript levels of each *Meg* gene in the BETC, PE and SEC. The six highly-expressed genes are highlighted in green. Note that *Meg* transcripts are detected exclusively in BETC. **(C)** Abundances of Meg proteins in the maize endosperm at three developmental stages. The histogram is based on results from searching the maizeproteome.ucsd.edu. Meg proteins not found in the proteome database are omitted from the histogram. The x-axis is scaled to the normalized arbitrary unit according to the maize proteome database.

To determine transcription profiles of *Meg* genes in the endosperm, we measured mRNA levels from basal endosperm transfer cells (BETCs), starchy endosperm cells (SECs) and peripheral endosperm (PE) containing aleurone cells at three developmental stages (Figure 5A). We found that the transcript levels of six *Meg* genes (*Meg1*, *Meg2, Meg4, Meg6, Meg9*, and *Meg13*) are significantly higher than those of other *Meg* genes (Unpaired *t* test: two-tailed *p* < 0.0001) (Figure 5B). These genes are all highly expressed specifically in BETCs at 8, 12 and 16 days after pollination (DAP) (FPKM > 4800), with the three consecutive *Meg* genes, *Meg2, Meg6* and *Meg9* being the most highly transcribed (Figure 5B). In contrast to these highly-expressed *Meg* homologs, five *Meg* genes showed negligible transcription levels across all cell types and time points (*Meg7, Meg8, Meg3, Meg10* and *Meg14*, FPKM < 365), and the two remaining *Meg* genes had intermediate levels of transcription, specifically in BETCs (FPKM = 1368 and 1910 for *Meg9* and *Meg11*, respectively).

These differences in the transcript levels of *Meg* genes correlate well with preservation of gene integrity in the *Meg* genes. The promoter and/or the two canonical exons are disrupted in the five *Meg* genes with low FPKM values (Figure 1). *Meg11* and *Meg12* exhibit intermediate transcript levels and appear to have the canonical *Meg* gene structure. However, *Meg11* has a 22 bp deletion in its promoter, and *Meg12* contains a frame shift mutation, which may affect the stability of its transcript. *Meg12* has been annotated as a pseudogene (www.maizesequences.org).

Despite the large variation in transcript levels, all *Meg* genes displayed similar spatiotemporal expression patterns. Their transcripts were strictly confined to BETCs, and transcription levels were highest at 8 DAP, but decreased thereafter (Figure 5B). These results suggest that the expansion of the *Meg* gene family in maize does not include diversification of expression patterns but does include variation in expression level across homologs, with more recently-derived intact genes generally having higher expression levels.

To further examine expression of *Meg* genes at the protein level, we searched the Atlas of Maize Proteotypes database (http://maizeproteome.ucsd.edu), where results from proteomic analyses of maize seed tissues are cataloged. Peptides were identified from six *Meg* genes, corresponding to the six genes with the highest transcript concentrations in the endosperm (Figure 5C). Peptides from the other 7 *Meg* genes were absent from the database. Furthermore, the protein abundance of highly-expressed *Meg* genes peaked at 8–10 DAP and reduced thereafter, in agreement with their transcript levels.

**Figure 6:**
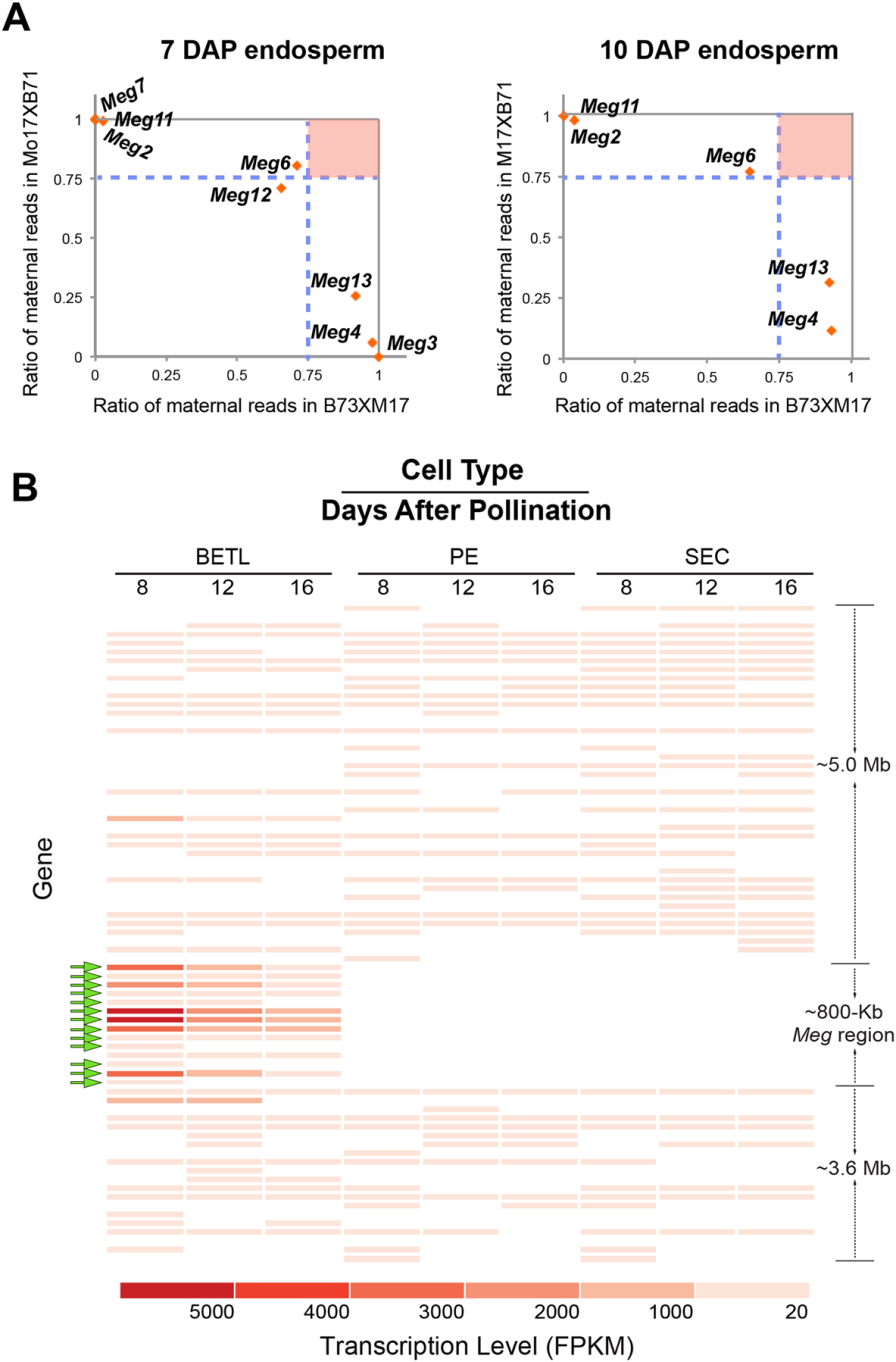
**Imprinting status of*Meg*genes and endosperm expression patterns of non-*Meg*genes in the*Meg*region. (A)** Maternal expression ratios of *Meg* genes at 7 DAP (left panel) and 10 DAP (right panel) endosperms from B73XM17 reciprocal crosses. The horizontal and vertical dotted lines mark boundaries of 3:1 maternal and paternal expression ratio in each cross. If the maternal allele of a gene is expressed 3 times more than its paternal allele, the gene should appear in the upper right corner (red square). The ratios were calculated from the endosperm transcriptome data by Xin *et al.*[39]. Expression of Meg genes was not detected in 15 DAP endosperm. **(B)** Heat map depicting the transcriptional activities in BETCs of genes within a ~9.4-Mb region spanning the *Meg* gene cluster in. Normalized gene expression level (FPKM) was used to generate the graphic. *Meg* genes are marked with green arrows. The FPKM values of the 6 highly-expressed *Meg* genes are far larger (>3000) than those of any other genes in the 9.4 Mb interval. Genes with FPKM < 20 in any of the nine samples were omitted from the heat map.

Because *Meg1* is a maternally expressed imprinted gene, we examined imprinting status of other *Meg* genes from publicly available transcriptome datasets generated by reciprocal crosses of B73XMo17 [38]–[40]. *Meg1* expression is maternally imprinted at 4 DAP but it becomes biallelic at 12 DAP [14]. The transcriptome datasets were generated from endosperm samples at 7 DAP and 10 DAP, before *Meg1*’s imprinted expression disappears. First, we compared coding sequences of all *Meg* genes to determine their single nucleotide polymorphisms (SNPs) in B73 and in Mo17 inbred lines. We were able to identify SNPs in 8 *Meg* alleles of B73 and Mo17 (Additional file 3: Figure S4) and maternal to paternal expression ratios of the 8 genes were available in the dataset by Xin *et al.*[39]. Unlike *Meg1*, none of the 8 genes exhibited parent-of-origin specific expression. Instead, Mo17 alleles of *Meg2*, *Meg7*, and *Meg11* displayed strong dominance over those of B73 while B73 alleles of *Meg3*, *Meg4*, and *Meg13* overwhelmed those of Mo17 (Figure 6A). *Meg6* and *Meg12* did not exhibit allele specific expression patterns. No SNPs were identified in B73 and Mo17 alleles of *Meg1*, *Meg3*, *Meg9* and *Meg10* and we were not able to find information about their parent of origin specific expression in the datasets. Expression data of *Meg3, Meg4, and Meg13* were available from Waters *et al.*[38] and they were consistent with the results in Figure 6A. These suggest that parent-of-origin specific expression of *Meg1* is not conserved in the 8 *Meg* duplicates that we examined in the B73XMo17 expression datasets.

The *Meg* gene region comprises 48 annotations in the B73 genome database (AGPv2, working gene set), including the 13 *Meg* genes. Among the 35 other annotations, 13 are transposable elements, 11 are pseudogenes or devoid of coding sequences, and 11 are predicted to be protein-coding genes with intact open reading frames. To determine whether the 11 putative protein-coding genes are transcriptionally active in the endosperm, we searched our endosperm transcriptome data using the BLAST program. Transcripts from three genes (GRMZM2G553132, GRMZM2G144653, GRMZM2G150091) were identified as transcribed in endosperm, but their levels ranged from 5% to 20% of the *Meg6* transcript (Figures 2B, 6B). GRMZM2G144653 is expressed in all three cell-types, while GRMZM2G553132 and GRMZM2G150091 are expressed specifically in BETCs. The high levels of *Meg* transcripts in BETCs suggest that the *Meg* region corresponds to a transcriptional “hotspot” in BETCs, even though the region exhibits features of pericentromeric heterochromatin.

## Conclusions

The *Meg* gene family has expanded radically in maize since its divergence from sorghum. However, the functional consequences of this expansion remain unclear. Meg proteins are members of the CRP superfamily, other members of which play diverse roles in cell signaling and defense in eukaryotic cells [3]. Most maize *Meg* genes are expressed exclusively in the BETL, and it is evident that *Meg1* is involved in the control of nutrient transport by promoting BETL formation [20]. Both sorghum and maize have BETLs [41],[42], but *Meg* genes have expanded only in maize. This suggests that the cell-signaling networks controlling seed development and nutrient allocation through the BETL may have diversified in maize. Alternatively, *Meg* gene-family expansion could function to alter the molecular mechanisms responsible for isolating the developing seed from infections in the maternal tissue in maize. The loss of imprinting in *Meg* genes is in line with the notion that functional diversity in the *Meg* family expanded along its evolution. Further examination of the functional roles played by *Meg* family genes is likely to enhance our understanding of how tandem gene duplication events contribute to species-specific adaptation in plants.

In this study, we examined the evolution of recently-duplicated genes to identify molecular selection by the combined use of phylogenetic and population-genetic analyses and to identify functional differences between duplicates by characterizing their expression, localization, imprinting, and protein structures. We observed changes in coding exons and promoter sequences throughout the *Meg* gene array in maize, consistent with a model in which mistakes introduced during the production of tandemly-duplicated gene arrays may be an important source of differences in both gene expression and protein function. We expect that a thorough understanding of gene duplication processes will illuminate the potential roles of “imperfect” gene duplication in generating the molecular diversity necessary to drive evolution and adaptation.

## Methods

### Cell type-specific sample preparation and sequencing library construction

It was impossible to differentiate transcription levels of each *Meg* gene by RNA blot analysis or by quantitative RT-PCR due to their high nucleotide sequence similarities. We determined nucleotide polymorphisms in the two exons present in all *Meg* genes and differentiated Illumina reads that mapped to the exons of different *Meg* genes according to their sequence polymorphisms (Additional file 3: Figure S4). Therefore, we used Illumina sequencing technology to identify and quantify transcripts of each member of the *Meg* family. Three endosperm cell types were isolated and total RNA samples from each cell type were prepared as described in [21],[43]. In brief, maize B73 kernels at 8, 12, and 16 DAPs were frozen in liquid nitrogen and mounted on the specimen holder with Tissue-Tek OCT compound (Ted Pella, CA). 50-μm sections were collected at −15°C and the frozen sections were dehydrated in ethanol and stained with the HistoGene LCM frozen section staining kit (MDS Analytical Technologies, CA). BETL, SEC, and PE samples were cut out from the sections using surgical scalpels under a dissecting stereomicroscope until we acquired approximately 2 μg total RNA for each sample. Total RNA samples were purified with the Arcturus PicoPure RNA isolation kit (Life Technologies, CA) and RNA-seq libraries were constructed according to a previously published protocol by Zhong *et al.*[43], with slight modifications. First-strand cDNA was generated using random hexamer-primed reverse transcription, and second strand cDNA synthesis and adaptor ligation were subsequently performed. cDNA fragments of approximately 200–500 bp were isolated by Ampure XP (Beckman Coulter) and cDNA fragments were amplified by 15 cycles of PCR. The library was quantified by quantitative PCR and by a bioanalyzer (Agilent, CA) before being sequenced on an Illumina HiSeq 2000 platform. The bright-field micrograph in Figure 5 was prepared as described in [44]. The Illumina reads have been deposited at NCBI Sequence Read Archive (Accession numbers SRA175303).

### Sequence preprocessing, mapping, and quantification of gene expression

The raw data were grouped into separate files by the barcode. The adapter sequence was removed using CUTADAPT [45],[46] followed by barcode trimming, quality trimming (−t 20, −l 50), artifact removal, and quality filtering (−q 20, −p 90) using FASTX-TOOLKIT (http://hannonlab.cshl.edu/fastx_toolkit/). Mitochondrial, chloroplast and ribosomal reads were removed by bowtie2 [47] using the default setting. The processed reads were put back into the paired end mode, or single end mode, if only one end was left.

Reads were aligned using GSNAP [48] with the supplied known splice junction from RefGen_v2 working gene set, with the parameters: −-nthreads 12, −-batch 5, −-max-mismatches 0.05, −-npaths 5, −-quiet-if-excessive, −-novelsplicing yes, −-split-output. Only reads in the files concordant_uniq, and concordant_mult, halfmapping_uniq, unpaired_uniq and unpaired_mult were combined for the downstream analysis. During this process, the translocation, scramble and inversion reads were excluded from downstream analysis. PICARD (http://picard.sourceforge.net/) was used to remove the duplicate reads in the combined alignment for each sample.

Final cleaned alignments were assembled using CUFFLINKS [49] with parameters --multi-read-correct, −-max-intron-length 8000, −-min-intron-length 20, −-GTF ZmB73_5a_WGS-chr1-10.gff, −b ZmB73_5b-chr1-10.fasta. Expression levels measured by FPKM (fragments per kilobase of exon per million fragments mapped) [49] were extracted for each member identified as being in the *Meg* gene family based on the CUFFLINKS results. For imprinting analyses, datasets from Xin *et al.*[39], Waters *et al.*[38], and Zhang *et al.*[40] were obtained from the Plant Cell website and each *Meg* gene was searched for its expression ratio from reciprocal crosses.

### Sequence annotation

Gene density in maize inbred line B73 was estimated based on the size of the maize genomic assembly (version AGPv2) and the total number of filtered genes (version 5b) (http://www.maizesequence.org/). Transposable elements (TEs) were identified by searching the Maize transposable element (TE) database (http://maizetedb.org/) using BLASTN program with an E-value cutoff of 1E-20.

### Phylogenetic reconstructions

The translated amino acids of *Meg* genes were aligned in MUSCLE [50], followed by manual corrections, and the protein-based alignment was then used to construct DNA alignments. Maximum likelihood phylogenies were constructed using Phyml v3.0 [51], with the evolutionary model selected by Akaike information criterion (AIC) [52]. Best-fit evolutionary models were JTT + G + F for protein sequences and HKY + G for nucleotide alignments. Clade support was inferred using SH-like approximate likelihood ratio tests (aLRTs) [28].

### Protein-coding adaptation

We inferred protein-coding adaptation using a branch-specific model to infer branches with an excess of positive selection, implemented in PAML v4.5 [30],[53]. Model M2a (positive selection) was applied to each branch on the phylogeny and compared to model M1a (nearly neutral) to identify specific branches undergoing positive protein-coding adaptation. Significance was assessed using a chi-square test with 2 degrees of freedom [54]. We corrected for multiple testing using a Bonferroni correction; we report only those results identified as adaptive at *p* < 0.05, after correcting for multiple tests. Adaptation was further localized to specific positions using branch-sites analysis (Zhang *et al*) [30], with positions having posterior probability >0.95 being inferred as adaptive.

### Secondary structure prediction

Consensus secondary structure was predicted and generated on the Network sequence analysis server [55] (NPS@, Network Protein Sequence Analysis, http://npsa-pbil.ibcp.fr/). Many algorithms for predicting protein secondary structures, such as hierarchical neural network, double prediction method, discrimination of protein secondary structure class, Garnier, Gibrat, multivariate linear regression combination, PHD, Predator, and SOPM on the NPS@ server were utilized.

### Identification of selective sweeps

Selective sweeps were identified using single nucleotide polymorphism (SNP) data from the maize HapMap database (http://www.panzea.org) (Chia *et al*; Hufford *et al*) [35],[36]. We excised the 30-Mb region of genomic DNA surrounding the *Meg* gene cluster and assessed support for an adaptive sweep using a composite likelihood ratio test (CLRT) (Nielsen *et al*) [37]. The CLRT calculates the likelihood of the local site frequency spectrum (SFS) at a specific location in the genome under two models: 1) the background SFS calculated across the entire region and 2) a one-parameter model that induces a characteristic sweep-like skew in the background SFS. Support for the sweep model is reported as the log-likelihood ratio of the sweep model to the background SFS. We scanned the 30-Mb genomic region for adaptive sweeps sampling every 100 bp.

Significance was assessed using 100,000 coalescent simulations under a standard-neutral model, simulated conditional on the observed number of segregating sites and pattern of sequencing coverage in each region. For each simulated replicate dataset, we calculated the log-likelihood ratio in favor of an adaptive sweep using the CLRT, producing a null distribution from which to estimate the *P-*value of the observed log-likelihood ratio. This approach has been shown to be robust to changes in demographic history such as population bottlenecks (Nielsen et al. [37]).

## Competing interests

The authors declare that they have no competing interest.

## Authors’ contribution

YX and B-HK designed this study. YX acquired most of data. YX, WM, EK, HH, BB, SB, and B-HK analyzed the *Meg* loci, their genomic region, and expression data. YX, KM, BK, and B-HK performed evolutionary analyses. YX, BK, and B-HK wrote the manuscript. All authors read and approved the final manuscript.

## Additional files

## Supplementary Material

Additional file 1: Table S1. Members of the *Meg* gene family. Click here for file

Additional file 2: Table S2. Locally duplicated gene families in Maize. Click here for file

Additional file 3: **Syntenic relationship of the Meg region with homologous regions of sorghum chromosome 2, rice chromosome 7, and maize chromosomes 2 and 7.** Homologous and homeologous regions of the *Meg* cluster were identified by the GEvo analysis (Lyons and Freeling, 2008). *Meg* gene models are depicted by red bars while all other genes are indicated with green bars. Both the sorghum chromosome 2 and maize chromosome 7 contain many copies of F-box genes in this region. The F-box genes in the two regions are connected with blue lines. All other anchor genes are connected with gray lines. All gene models and intervals were drawn to scale according to their physical sizes. The figure was adapted from the GEvo analysis results. Click here for file
