# Supplementary material for: Adaptive expansion of the maize maternally expressed gene (Meg) family involves changes in expression patterns and protein secondary structures of its members
Source: BMC Plant Biol. 2014 Aug 1;14:204. doi: 10.1186/s12870-014-0204-8 (PMC4236715; doi:10.1186/s12870-014-0204-8)
Supplement: Additional file 1: Table S1. — Members of the Meg gene family. [file s12870-014-0204-8-S1.docx]

**Table S1. Members of the *Meg* gene family**

| Locus | Gene name | | | Coordinate | | | |  | | 5' retrotransposon | | | |  | | 3' DNA transposon | | | |
| --- | --- | --- | --- | --- | --- | --- | --- | --- | --- | --- | --- | --- | --- | --- | --- | --- | --- | --- | --- |
|  |  |  |  | Start | | end | |  | | Name | | bp* | |  | | Name | | bp** | |
| GRMZM2G354335 | | *Meg1* | 13,065,608 | | 13,066,906 | |  | | *prem1* | | 622 | |  | | *CACTA* | | 901 | |  |
| GRMZM2G116212 | | *Meg7* | 12,995,597 | | 12,996,311 | |  | | *ji* | | 0 | |  | | *CACTA* | | 734 | |  |
| GRMZM2G123153 | | *Meg8* | 12,851,417 | | 12,851,623 | |  | | *xilon-diguus* | | 160 | |  | | *CACTA* | | 894 | |  |
| GRMZM2G344323 | | *Meg3* | 12,768,363 | | 12,771,318 | |  | | *xilon-diguus* | | 313 | |  | | *CACTA* | | 807 | |  |
| GRMZM2G502035 | | *Meg2* | 12,744,197 | | 12,744,826 | |  | | *xilon-diguus* | | 484 | |  | | *CACTA* | | 806 | |  |
| GRMZM2G094054 | | *Meg6* | 12,701,416 | | 12,702,254 | |  | | *xilon-diguus* | | 314 | |  | | *CACTA* | | 721 | |  |
| GRMZM2G088896 | | *Meg9* | 12,668,551 | | 12,669,739 | |  | | *xilon-diguus* | | 39 | |  | | *CACTA* | | 724 | |  |
| GRMZM2G086827 | | *Meg10* | 12,665,864 | | 12,666,177 | |  | | *xilon-diguus* | | 307 | |  | | *CACTA* | | 807 | |  |
| GRMZM2G181051 | | *Meg11* | 12,618,639 | | 12,619,361 | |  | | *xilon-diguus* | | 226 | |  | | *CACTA* | | 731 | |  |
| GRMZM2G137959 | | *Meg4* | 12,583,271 | | 12,584,140 | |  | | *xilon-diguus* | | 223 | |  | | *CACTA* | | 733 | |  |
| GRMZM2G175896 | | *Meg12* | 12,526,620 | | 12,527,455 | |  | | *xilon-diguus* | | 316 | |  | | *CACTA* | | 725 | |  |
| GRMZM2G175912 | | *Meg13* | 12,479,307 | | 12,480,176 | |  | | *prem1* | | 344 | |  | | *CACTA* | | 707 | |  |
| GRMZM2G145466 | | *Meg14* | 13,170,216 | | 13,171,003 | |  | | *ji* | | 2072 | |  | |  | | - | |  |

* These numbers indicate the distance (base pairs) from the 3' end of a retrotransposon to the 5' end of the corresponding downstream *Meg* gene.

** These numbers indicate the distances (base pairs) from the 3' end of a *Meg* gene to the 5' end of the corresponding downstream *CACTA* DNA transposon

The 8 new *Meg* members are printed in blue in the second column.
