# Supplementary material for: Adaptive expansion of the maize maternally expressed gene (Meg) family involves changes in expression patterns and protein secondary structures of its members
Source: BMC Plant Biol. 2014 Aug 1;14:204. doi: 10.1186/s12870-014-0204-8 (PMC4236715; doi:10.1186/s12870-014-0204-8)
Supplement: Additional file 2: Table S2. — Locally duplicated gene families in Maize. [file s12870-014-0204-8-S2.docx]

**Table S2. Locally duplicated gene families in Maize**

| **Locus** | **Duplication**  **type** | **Repeat size (gene size) (kb)** | **# of repeats** | **region size (kb)** | **Reference** | **Protein** |
| --- | --- | --- | --- | --- | --- | --- |
| Prolamin gene/gamma | Tandem | 12 (3.2-3.4) | 2 | 30 | Das et al, 1991 | Storage protein |
| Prolamin gene/alpha A1 | Proximal/ Tandem | ? (0.70) | 12 | 140 | Xu et al, 2008 | Storage protein |
| Prolamin gene/alpha B | Proximal/ Tandem | ?( 0.72) | 9 | 220 | Xu et al, 2008 | Storage protein |
| Prolamin gene/alpha C | Tandem | ? (0.80) | 16 | 140 | Xu et al, 2008 | Storage protein |
| Prolamin gene/alpha D | Proximal/ Tandem | ? (0.72) | 5 | 240 | Xu et al, 2008 | Storage protein |
| *kn1* | Tandem | 17 (8) | 2 | 34 | Lowe et al, 1992 | homeodomain protein |
| *pl1* | Tandem | ? (~2.5) | 3 | 22 | Pilu et al, 2003 | Transcription factor |
| *a1-b* | Proximal | 4.2-5.2 (1.7-8.2) | 2 | <=75 | Yandeau-Nelson et al, 2006 | NADPH-dependent reductase |
| *R-r* | Proximal/ Tandem | ? (3) | 4 |  | Walker et al, 1995 | HLH type transcription factor |
| *R-st* | Tandem | 14 ? (2.3?) | 4 |  | Eggleston et al, 1995 | HLH type/Class III (myc) |
| *rp1* | Proximal/ Tandem (most) | ~1.5-4 (~1.5-4) | 8 | 300 | Ramakrishna et al, 2002 | NBS-LRR protein |
|  |  | ~1.5 (~1.5) | 7 | 250 |  |  |
| *rp3* | N/A |  | 5 (B73) | 140 | Webb et al, 2002 | NBS-LRR protein |
| *p1* | Tandem | 12.6-13.0 (12.6-13.0) | 11 | 380 | Goettel et al, 2009 | R2R3 Myb-like |
| *meg1* | Proximal | 1.3-8.9 (0.8) | 13 | 800 |  |  |

References

Das OP, Ward K, Ray S and Messing J. 1991. Sequence variation between alleles reveals two types of copy correction at the 27-kDa zein locus of maize. Genomics 11: 849-856.

Eggleston WB, Alleman M and Kermicle JL. 1995. Molecular organization and germinal instability of R-stippled maize. Genetics 141: 347-360.

Goettel W and Messing J. 2009. Change of gene structure and function by non-homologous end-joining, homologous recombination, and transposition of DNA. PLoS Genetics 5: e1000516. doi: 10.1371/journal.pgen.1000516

Lowe B, Mathern J and Hake S. 1992. Active Mutator elements suppress the knotted phenotype and increase recombination at the Kn1-O tandem duplication. Genetics 132: 813-822.

Lyons E and Freeling M. 2008. How to usefully compare homologous plant genes and chromosomes as DNA sequences. Plant Journal 53: 661-673. doi: 10.1111/j.1365-313X.2007.03326.x

Pilu RR, Piazza PP, Tonelli CC and 6. 2003. pl-bol3, a complex allele of the anthocyanin regulatory pl1 locus that arose in a naturally occurring maize population. Plant Journal 36: 510-521. doi: 10.1046/j.1365-313X.2003.01898.x

Ramakrishna W, Emberton J, Ogden M, SanMiguel P and Bennetzen JL. 2002. Structural analysis of the maize rp1 complex reveals numerous sites and unexpected mechanisms of local rearrangement. Plant Cell 14: 3213-3223.

Walker EL, Robbins TP, Bureau TE, Kermicle J and Dellaporta SL. 1995. Transposon-mediated chromosomal rearrangements and gene duplications in the formation of the maize R-r complex. EMBO J 14: 2350-2363.

Webb CAC, Richter TET, Hulbert SHS and 7. 2002. Genetic and molecular characterization of the maize rp3 rust resistance locus. Genetics 162: 381-394.

Xu JH and Messing J. 2008. Organization of the prolamin gene family provides insight into the evolution of the maize genome and gene duplications in grass species. Proc Natl Acad Sci USA 105: 14330-14335. doi: 10.1073/pnas.0807026105

Yandeau-Nelson MD. 2006. Unequal Sister Chromatid and Homolog Recombination at a Tandem Duplication of the a1 Locus in Maize. Genetics 173: 2211-2226. doi: 10.1534/genetics.105.052712
