# Supplementary material for: Adaptive expansion of the maize maternally expressed gene (Meg) family involves changes in expression patterns and protein secondary structures of its members
Source: BMC Plant Biol. 2014 Aug 1;14:204. doi: 10.1186/s12870-014-0204-8 (PMC4236715; doi:10.1186/s12870-014-0204-8)
Supplement: Additional file 3: — Syntenic relationship of the Meg region with homologous regions of sorghum chromosome 2, rice chromosome 7, and maize chromosomes 2 and 7. Homologous and homeologous regions of the Meg cluster were identified by the GEvo analysis (Lyons and Freeling, 2008). Meg gene models are depicted by red bars while all other genes are indicated with green bars. Both the sorghum chromosome 2 and maize chromosome 7 contain many copies of F-box genes in this region. The F-box genes in the two regions are connected with blue lines. All other anchor genes are connected with gray lines. All gene models and intervals were drawn to scale according to their physical sizes. The figure was adapted from the GEvo analysis results. [file s12870-014-0204-8-S3.docx]

**Additional Files**


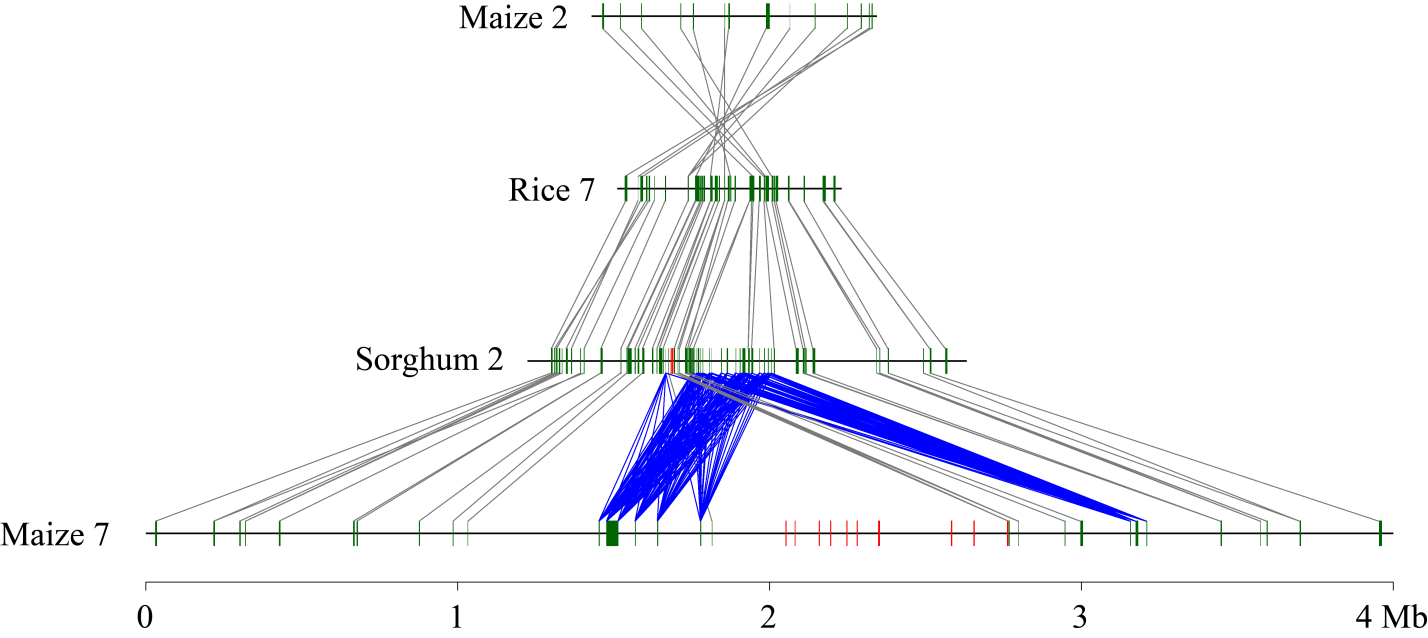


**Figure S1.** Syntenic relationship of the Meg region with homologous regions of sorghum chromosome 2, rice chromosome 7, and maize chromosomes 2 and 7.

Homologous and homeologous regions of the *Meg* cluster were identified by the GEvo analysis (Lyons and Freeling, 2008). *Meg* gene models are depicted by red bars while all other genes are indicated with green bars. Both the sorghum chromosome 2 and maize chromosome 7 contain many copies of F-box genes in this region. The F-box genes in the two regions are connected with blue lines. All other anchor genes are connected with gray lines. All gene models and intervals were drawn to scale according to their physical sizes. The figure was adapted from the GEvo analysis results.

**
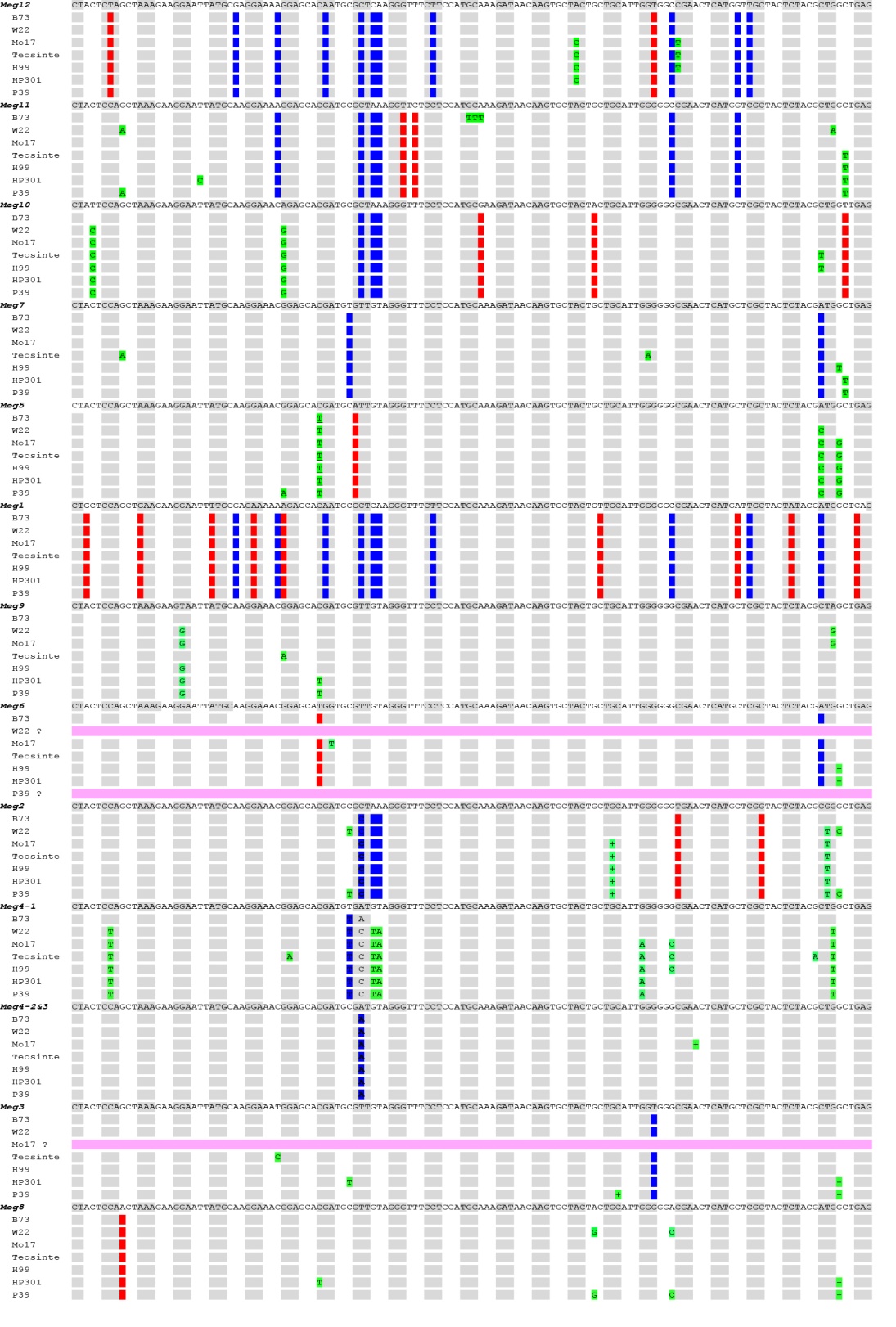
**

**Figure S2.** Estimation of *Meg* copy numbers in six maize cultivars and in teosinte

Sequence polymorphisms in 6 maize inbred lines and in teosinte (*Zea mays, subsp parviglumis*) were determined with the Ion Torrent amplicon sequencing method. Each orthologous copy was differentiated by unique combinations of polymorphic sites in the *Meg* family. The polymorphic sites were identified as described in Supplemental Figure 4. The red squares mark the loci-specific sites and the blue squares correspond to the sites shared by two or more loci. The B73 sequence of each locus is shown next to the gene name. The two canonical exons were amplified and sequenced (see Supplemental Figure 4). The primer sequences for amplifying the conserved exons are GTCACAGATGATGTCGGTGTTTCT and TTAGAAGCAGGCATGACTACACT.


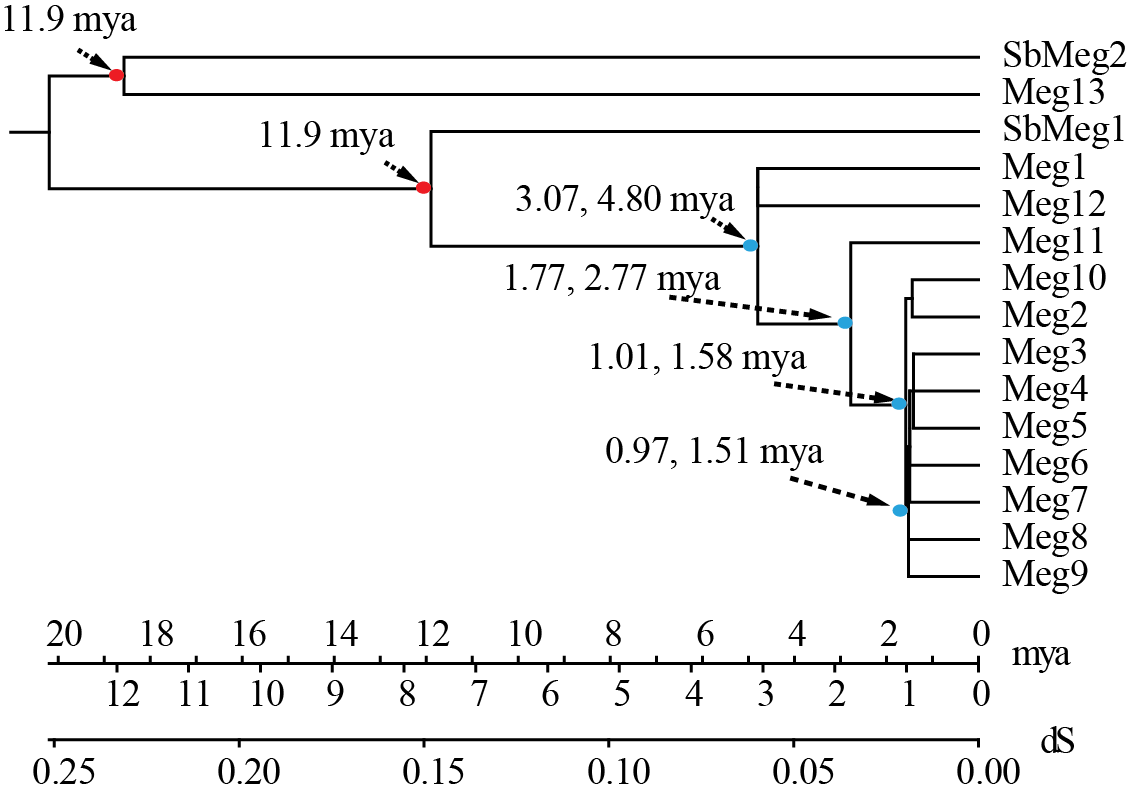


**Figure S3.** Maximum likelihood phylogenetic tree under a molecular clock.  The synonymous substitutions per synonymous site (dS) were estimated using the ML method implemented in the Codeml program of PAML version 4.4. The free-ratio “branch model” (model = 1, CodonFreq = 3) were used, which allowed the calculation of a distinct ratio for each branch. CodonFreq=3 represents free parameters of equilibrium codon frequencies. The maize-sorghum divergence date (11.9 mya) was used to calibrate the two branches representing the two common ancestral *Meg* genes in the common ancestor of maize and sorghum. The predicted speciation event was marked with red points. Two evolutionary time scales (in millions of years ago, mya) representing the two calibrated age based on the different common ancestor branches. Two ages were assigned to the major gene duplication events according to the two dating scheme.

**
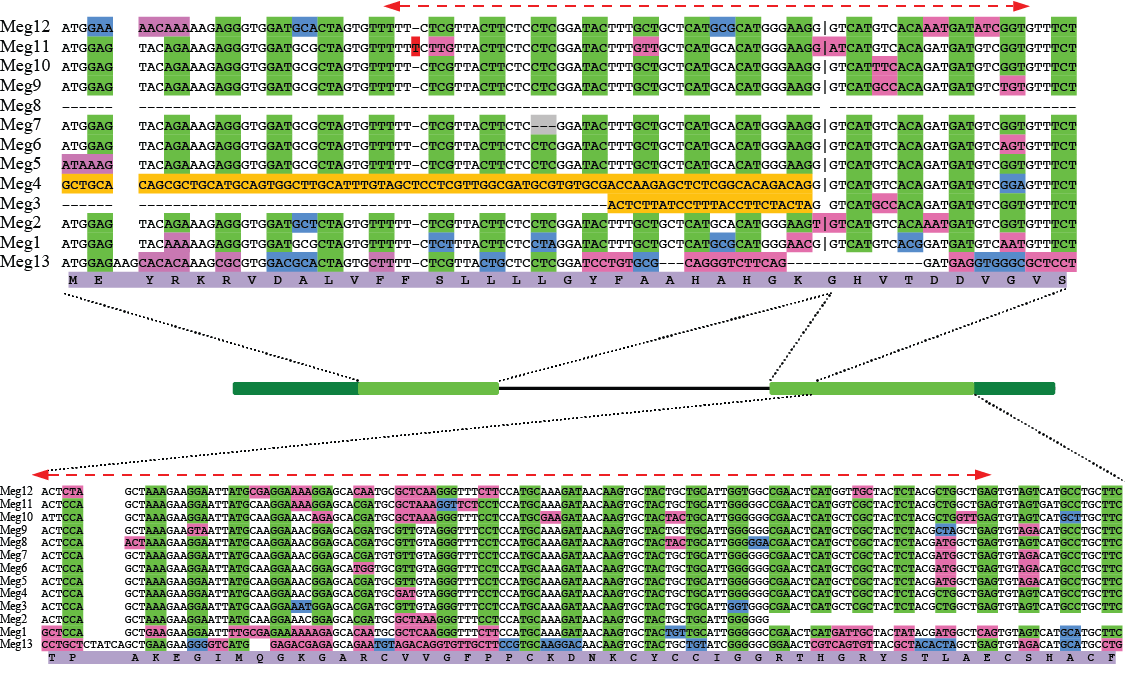
**

**Figure S4.** Single nucleotide polymorphisms in the *Meg* family

The nucleotide alignments of the two conserved exons of *Meg* genes that comprise the entire coding region. The gene structure of the *Meg* loci is illustrated in the middle. The red dashed lines mark the regions that were amplified and analyzed by sequencing with the Ion Torrent technology to detect orthologous *Meg* loci in maize inbred lines and in teosinte. The synonymous and nonsynonymous substituted codons are shaded in blue and red, respectively. The nucleotides highlighted in yellow indicate non-homologous regions. The polymorphisms are determined by comparing to a consensus sequence of all 13 loci of the *Meg* family in the maize B73 genome. The consensus sequence was inferred by nucleotides that are conserved in more than 7 loci.

**References for table S2**

Das OP, Ward K, Ray S and Messing J. 1991. Sequence variation between alleles reveals two types of copy correction at the 27-kDa zein locus of maize. Genomics 11: 849-856.

Eggleston WB, Alleman M and Kermicle JL. 1995. Molecular organization and germinal instability of R-stippled maize. Genetics 141: 347-360.

Goettel W and Messing J. 2009. Change of gene structure and function by non-homologous end-joining, homologous recombination, and transposition of DNA. PLoS Genetics 5: e1000516. doi: 10.1371/journal.pgen.1000516

Lowe B, Mathern J and Hake S. 1992. Active Mutator elements suppress the knotted phenotype and increase recombination at the Kn1-O tandem duplication. Genetics 132: 813-822.

Lyons E and Freeling M. 2008. How to usefully compare homologous plant genes and chromosomes as DNA sequences. Plant Journal 53: 661-673. doi: 10.1111/j.1365-313X.2007.03326.x

Pilu RR, Piazza PP, Tonelli CC and 6. 2003. pl-bol3, a complex allele of the anthocyanin regulatory pl1 locus that arose in a naturally occurring maize population. Plant Journal 36: 510-521. doi: 10.1046/j.1365-313X.2003.01898.x

Ramakrishna W, Emberton J, Ogden M, SanMiguel P and Bennetzen JL. 2002. Structural analysis of the maize rp1 complex reveals numerous sites and unexpected mechanisms of local rearrangement. Plant Cell 14: 3213-3223.

Walker EL, Robbins TP, Bureau TE, Kermicle J and Dellaporta SL. 1995. Transposon-mediated chromosomal rearrangements and gene duplications in the formation of the maize R-r complex. EMBO J 14: 2350-2363.

Webb CAC, Richter TET, Hulbert SHS and 7. 2002. Genetic and molecular characterization of the maize rp3 rust resistance locus. Genetics 162: 381-394.

Xu JH and Messing J. 2008. Organization of the prolamin gene family provides insight into the evolution of the maize genome and gene duplications in grass species. Proc Natl Acad Sci USA 105: 14330-14335. doi: 10.1073/pnas.0807026105

Yandeau-Nelson MD. 2006. Unequal Sister Chromatid and Homolog Recombination at a Tandem Duplication of the a1 Locus in Maize. Genetics 173: 2211-2226. doi: 10.1534/genetics.105.052712
